# The Role of N6-Methyladenosine-Associated lncRNAs in the Immune Microenvironment and Prognosis of Colorectal Cancer

**DOI:** 10.1155/2022/4689396

**Published:** 2022-09-06

**Authors:** Congfei Yuan, Caidong Liu, Shuli Zhao, Xishan Zhang, Haifeng Jia, Baiyu Chen, Maojin Zhang, Yuan Zheng, Jin Zhou, Yanzhi Bo

**Affiliations:** ^1^Department of General Surgery, Lianshui County People's Hospital, Huai'an 223400, China; ^2^Department of Laboratory Medicine, Nanjing First Hospital, Nanjing Medical University, Nanjing 210006, China; ^3^General Clinical Research Center, Nanjing First Hospital, Nanjing Medical University, Nanjing 210006, China; ^4^Department of General Surgery, Nanjing First Hospital, Nanjing Medical University, Nanjing 210006, China

## Abstract

**Background:**

The role of N6-methyladenosine long noncoding RNAs (lncRNAs) in colorectal cancer (CRC) is elusive.

**Materials and Methods:**

We identified m6A-associated lncRNAs by using the data gathered from The Cancer Genome Atlas (TCGA) and stratified CRC patients into different subgroups. Cox regression analysis was performed to construct an m6A-associated lncRNA signature. The role of this signature in the immune microenvironment and prognosis was dissected subsequently. Finally, a gene set enrichment analysis (GSEA) was conducted to predict the possible mechanisms based on the signature.

**Results:**

Three m6A-associated clusters were constructed from 866 differentially expressed lncRNAs. Cluster 2 had poor prognosis and low immune cell infiltration. An m6A-associated lncRNA signature consisting of 14 lncRNAs was constructed and recognized as an independent prognostic indicator of CRC by using survival analysis and receiver operating characteristic (ROC) curves. The clinical features and immune cell infiltration status were significantly different in patients stratified by the risk score. Furthermore, GSEA showed that the P53 pathway and natural killer cell-mediated cytotoxicity were more enriched in the low-risk group.

**Conclusion:**

Our data revealed that m6A-associated lncRNAs could be potential prognostic indicators of immunogenicity in CRC.

## 1. Introduction

Colorectal cancer (CRC) is the third most prevalent gastrointestinal malignancy worldwide [1]. A significant number of CRC patients will ultimately relapse after curative treatments[2]. Hence, there is an urgent need to investigate prognostic markers for CRC.

N6-methyladenosine (M6A) is the most common posttranscriptional modification in RNAs [3]. Recent studies have indicated that m6A RNA modification plays an important role in biological processes and cancer pathogenesis [4]. Aberrant expressions of m6A regulators (e.g., METTL14, METTL3, KIA, ALKBH5, FTO, and YTHDF1/2/3) have been identified in numerous tumors [5–9]. A variety of pathological functions, ranging from tumor initiation, invasion, metastasis to tumor stem cell pluripotency, could be mediated by m6A methylation [10, 11]. Long noncoding RNAs (lncRNAs) are important epigenetic regulators that play critical roles in diverse physiological and pathological processes [12, 13]. Studies have reported that some lncRNAs participate in tumor initiation and progression [14–16]. Despite extensive efforts to define the pathogenesis of lncRNAs, the roles of lncRNAs in the m6A modification in CRC remain largely elusive.

Immune microenvironment has been found to be closely associated with the clinical outcome of immunotherapy and tumor development. In the present study, the coexpression network of the m6A-associated lncRNAs was investigated to obtain 68 m6A-associated prognostic lncRNAs. Then, we established three m6A-associated clusters in CRC, analyzed the characteristics of immune cell infiltration among tumor cells, and investigated whether m6A-associated lncRNAs clusters have prognosis values in CRC patients. Furthermore, we constructed a signature using 14 m6A-associated lncRNAs which could predict the prognosis of CRC patients.

## 2. Results

### 2.1. The Differential Expressions of m6A-Associated lncRNAs

A total of 19604 mRNAs and 14086 lncRNAs were screened from TCGA database. 1590 m6A-associated lncRNAs were obtained (/R/>0.4 and *p* < 0.05) according to 23 reported m6A-associated genes, of which 866 differentially expressed m6A-associated lncRNAs in CRC were detected with a log/fold change (FC)/>0.5 and a *p* < 0.001 (Supplementary data 1).

### 2.2. Identification of m6A-Associated lncRNAs with a Prognostic Value

**Figure 1:**
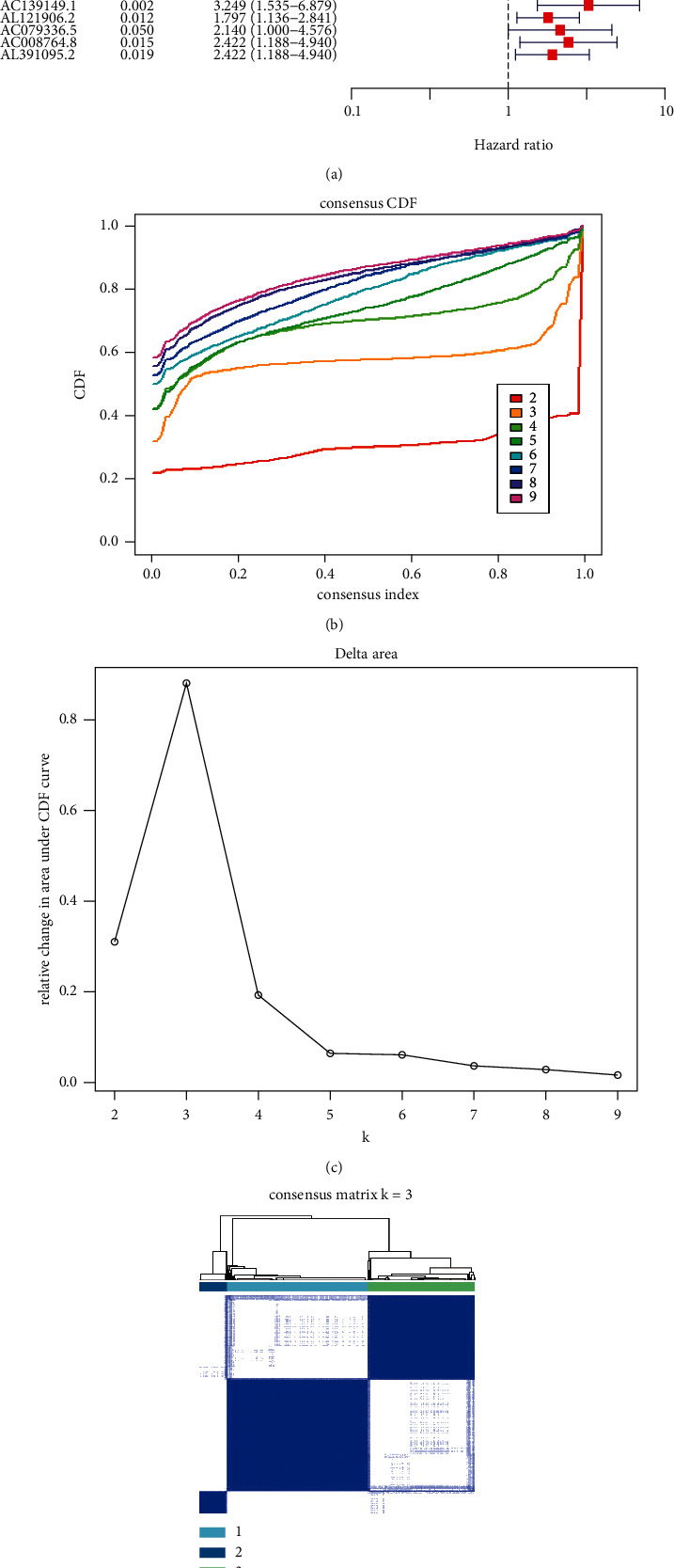
Unsupervised clustering of CRC using m6A-associated lncRNA expression data. (a). The forest plot of 68 prognostic m6A-associated lncRNAs. (b). Consensus Cumulative Distribution Function (CDF) curve of unsupervised clusters analysis. (c). Delta area under CDF curve of cluster analysis. (d). Cumulative distribution function graph of the consistency matrix at *K* = 3. The white and blue heatmap exhibits sample consensus. (e). Survival curve analysis of three clusters.

As shown in Figure 1(a), we annotated m6A-associated lncRNAs and clinical characteristics, then investigated the role of each lncRNA on the prognostic outcome of the patients with CRC. A total of 68 m6A-associated lncRNAs with obvious prognostic values were detected and used for further study.

### 2.3. Establishment of m6A-Associated lncRNA Clusters

To classify different m6A clusters based on lncRNAs, we mapped these 68 m6A-associated lncRNAs to expression profile of CRC samples to perform clustering using the Consensus Cluster Plus (CCP) tool. As shown in Figure 1(b), the number of clusters was sequentially set from 1 to 9, and CCP analysis indicated that the results were most stable when these m6A-related lncRNAs were separated into three clusters using the Consensus Cluster Plus *R* package (Figures 1(c), 1(d)). The OS data of each cluster was calculated using the Kaplan–Meier method, and the results displayed that there was significant difference among the survival of CRC patients in these three clusters (Figure 1(e)).

### 2.4. Clinical Characteristics and the Immune Score of Each Cluster in CRC

**Figure 2:**
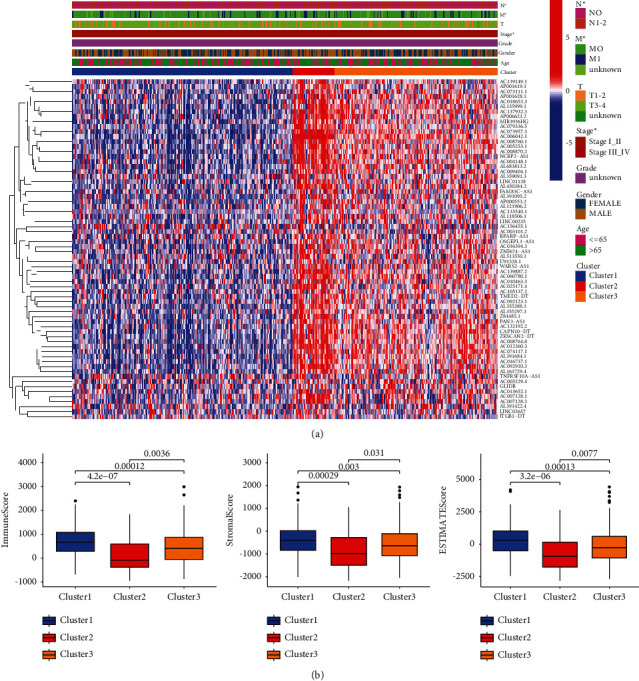
Clinical characteristics and immune score of m6A-associated lncRNAs in CRC. (a). Heatmap of the correlation between m6A-associated lncRNAs and clinical characteristics in the TCGA database. (b). Comparison of composition of immune score, stromal score and estimate score in cluster 1, 2 and 3.

As compared with cluster 1 and cluster 3, cluster 2 had the highest N stage, M stage, and TNM stage (Figure 2(a)). The ESTIMATE algorithm was employed to evaluate the accurate estimate score (tumor purity), immune score, and stromal score in accordance with the gene expression profiles of CRC patients. Our findings showed that compared with clusters 1 and 3, cluster 2 had the lowest estimate score, immune score, and stromal score (Figure 2(b)).

### 2.5. m6A-Associated lncRNAs Signature Construction

**Figure 3:**
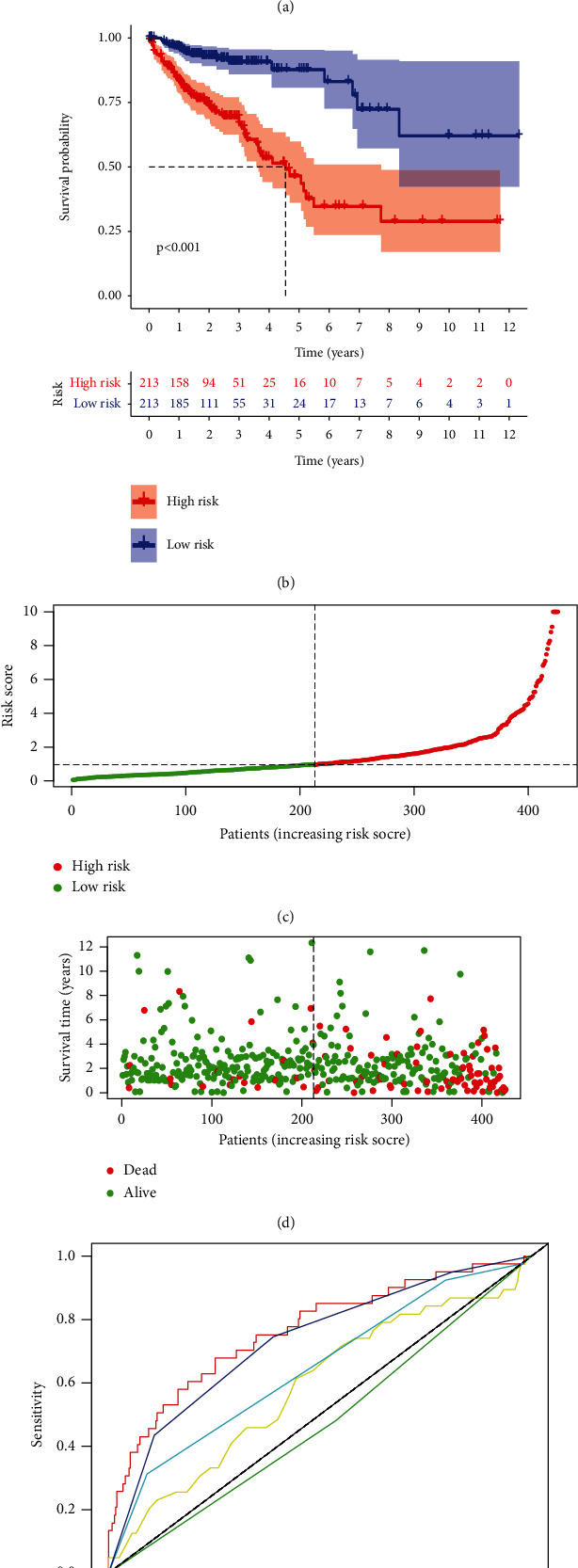
The signature based on m6A-associated lncRNAs for CRC patients. (a). The network of 14 m6A-associated lncRNAs. (b). Kaplan-Meier analysis of the low- and high-risk groups. (c-d). The distribution of risk scores and the survival state of selected m6A-associated lncRNAs. (e). The AUC of risk score and other clinicopathological factors. (f). The AUC for 1-, 3- and 5-year survival rates were 0.764, 0.743, 0.753, respectively.

**Figure 4:**
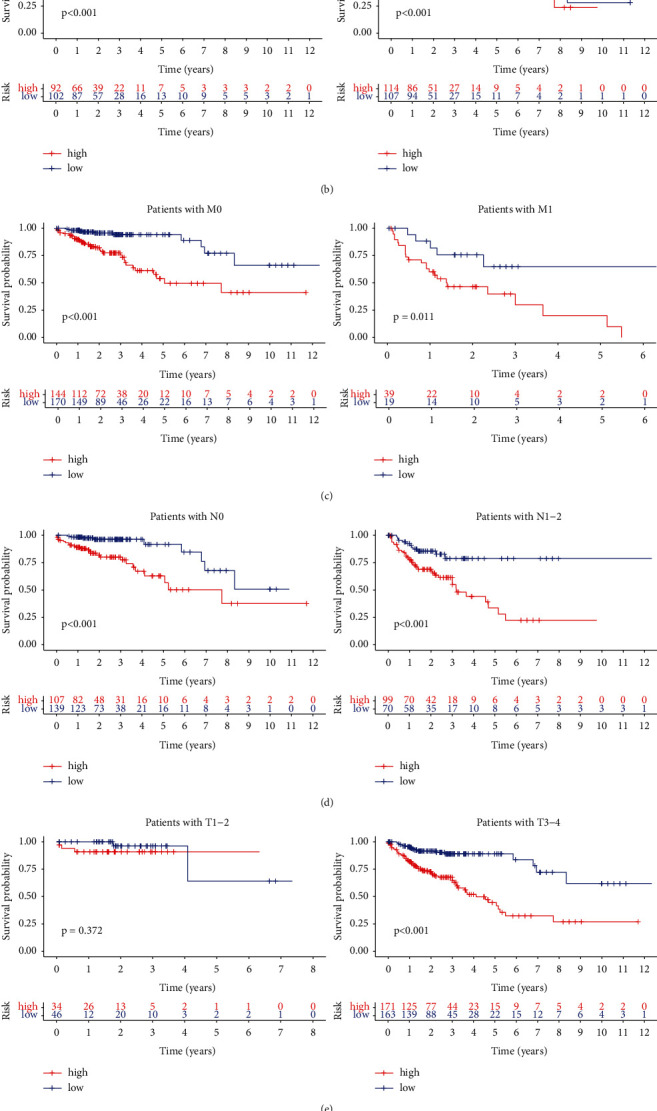
The prognostic value of the m6A-associated lncRNA signature in CRC patients. Kaplan-Meier analysis for the different risk groups classified using clinical factors including age (a), gender (b), M stage (c), N stage (d), T stage (e) and TNM stage (f).

As shown in Figure 3(a), a total of 14 m6A-associated lncRNAs that had a coexpression relationship with 8 m6A-associated genes were recognized as effective independent prognostic factors. Among them, AC137932.3, AL391422.4, AC092123.1, AC156455.1, AC132192.2, AC008760.1, RPARP-AS1, LINC02657, AP001619.1, AC003101.2, AL161729.4, TNFRSF10A-AS1, AL121906.2, and AC074117.1 were found to be favorable prognostic factors (Supplementary data 2). The risk score of each CRC patient = AC137932.3^*∗*^(−1.4041) + AL391422.4^*∗*^0.9484 + AC092123.1^*∗*^ (−1.3865) + AC156455.1^*∗*^0.1977 +  AC132192.2^*∗*^ (−0.4822) + AC0a08760.1^*∗*^0.5973 + RPARP-AS1^*∗*^0.3572 + LINC02657^*∗*^0.7205 + AP001619.1^*∗*^0.8025 + AC003101.2^*∗*^1.0959 + AL161729.4^*∗*^ 0.3047 + TNFRSF10A-AS1^*∗*^(−0.2329) + AL121906.2^*∗*^1.02629 + AC074117.1^*∗*^ 0.25582. Based on the median risk score, 426 CRC patients were classified into the low-risk and high-risk groups. The Kaplan–Meier curves and the distributions of survival status confirmed the poor outcome in the high-risk group (Figures 3(b)–3(d)). Our findings showed that the mortality was closely associated with the risk score. Moreover, the area under the curve (AUC) is measured, and the value for the prognostic risk score was 0.764 which is higher than AUCs of the other clinicopathological factors (Figure 4(e)). The AUC values corresponding to 1-, 3-, and 5-year of OS were 0.764, 0.743, and 0.753, respectively (Figure 3(f)). These data indicated the good prediction accuracy of this model.

### 2.6. The Validation of the Signature in CRC

The prognostic value of the m6A-associated lncRNA signature was investigated in CRC patients from TCGA dataset. The patients were classified by various clinical parameters, consisting of gender, age, T, N, M, and TNM stage. In almost all subgroups, the patients with a low-risk score trended to have a higher OS rate than that of the high-risk group (Figure 4).

**Figure 5:**
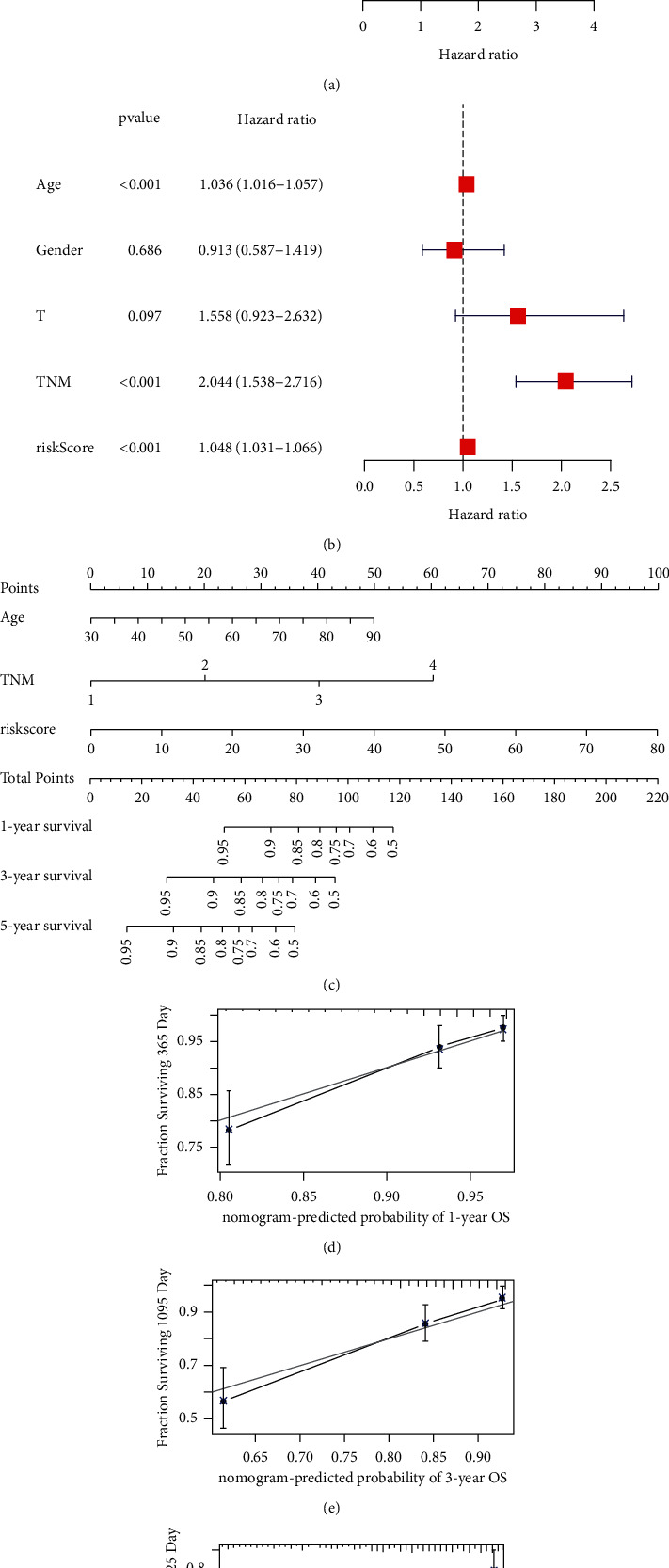
The independence and effectiveness of this model in predicting prognosis of CRC patients. Forest plots of univariate (a) and multivariate (b) Cox regression analysis in CRC. Nomogram model (c) to predict 1-, 3- and 5-year survival rates of CRC patients. Calibration graph showed the predicted 1- (d), 3- (e) and 5-year (f) survival rates were close to actual survival rates.

Next, we evaluated the independence and effectiveness of this model in predicting prognosis of CRC patients. Our findings showed that this m6A-associated lncRNA signature could be an effective and independent factor for predicting the outcome of the patients with CRC (Figures 5(a), 5(b)). Then, a nomogram was conducted to predict 1-, 3- and 5-year OS of the patients with CRC based on the results of univariate and multivariate Cox regression analyses, including age, TNM stage, and the risk score (Figure 5(c)). The calibration curves demonstrated well-prediction accuracy of this nomogram in CRC patients (Figures 5(d)–5(f)).

### 2.7. Gene Set Enrichment Analysis

**Figure 6:**
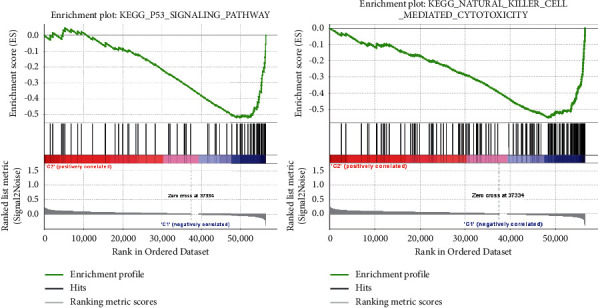
The enriched signaling pathways in the low-risk group. The GSEA results of the P53 signaling pathway and nature killer cells mediated cytotoxicity.

Finally, we evaluated the potential biological mechanisms associated with the risk model by GSEA. As shown in Figure 6, the P53 signaling pathway (NOM p-val = 0.0019, FDR q-val = 0.155) and natural killer cell-mediated cytotoxicity (NOM p-val = 0.0172, FDR q-val = 0.195) were more enriched in the low-risk group. Our study suggested that this risk-related model could be used for the personalized treatment of CRC patients.

## 3. Discussion

Previous studies have demonstrated the pivotal roles of m6A modification in various cancers including CRC [14–16]. Investigating the potential prognostic role of m6A-associated lncRNAs will facilitate understanding of the molecular mechanisms of CRC. In our work, 68 prognostic m6A-associated lncRNAs were identified, then three m6A-associated lncRNAs cluster groups were constructed using 426 CRC samples from TCGA database. Compared with cluster 1 and cluster 3, cluster 2 had the worst OS time and high pathological stage. In addition, ESTIMATE analyses revealed that the immune score was remarkably reduced in cluster 2. Our data suggested that m6A-associated lncRNAs might be used as a predictive biomarker.

It is generally known that there are currently some CRC prognostic indicators, including the TNM stage and tumor grade. However, more accurate prognostic factors are required to predict and analyze the OS rate in CRC patients. Current studies have indicated that lncRNAs play an important role in predicting the outcome and prognosis of various cancers. For instance, Yin, et al. [17] reported that overexpression of LINC01133 was related to the poor prognosis in patients with hepatocellular carcinoma. Feng, et al. [18] reported that lncRNA-CTS was aberrantly expressed in gastric cancer tissues, and the upregulation of CTS was closely associated with tumor volume, tumor histology, lymph node metastasis, and the poor prognosis. Recently, numerous m6A-associated lncRNAs are reported to be potential markers for the prediction of various cancers; Wang, et al. [2] established an 11 m6A-associated lncRNA signature and confirmed that it had a good prognostic value and could act as a valid marker for gastric cancer. Xu, et al. [19] established a risk model consisting of 12 m6A-associated lncRNAs and demonstrated that the model might be a promising prediction of prognosis in lung adenocarcinoma patients. In the present study, an m6A-associated lncRNA signature consists of 14 lncRNAs which could predict patients with poor prognosis. Moreover, we assessed the clinical value of the signature in gender, age, T, N, M, and TNM stage and identified that the signature was closely associated with the progression of CRC. Meanwhile, the GSEA analysis preliminary displayed that these lncRNAs were closely involved in the P53 pathway and NK cell-mediated cytotoxicity. Further studies are needed to demonstrate the mechanisms involved in this lncRNA signature.

## 4. Conclusion

In summary, our work defined a m6A-associated lncRNA signature which could predict the prognosis of CRC patients. This m6A-associated lncRNA signature will provide guidance for individualized treatment.

## 5. Methods

### 5.1. Data Acquisition and Processing of the CRC Dataset

The public RNA sequencing (RNA-seq) data from 512 patients' CRC were downloaded from TCGA (https://portal.gdc.cancer.gov/). Patients without survival information were removed.

### 5.2. Identification of m6A-Associated lncRNAs in CRC

The m6A-associated genes were gathered from TCGA database and selected based on the previously published articles [20, 21]. The m6A-associated lncRNAs were screened by Spearman correlation coefficient formula with|R| value > 0.6 and *p* value <0.001.

### 5.3. Consensus Clustering of m6A-Associated lncRNAs

On the basis of the expression levels of m6A-associated lncRNAs, the CRC patients were separately divided into three groups (clusters 1, 2, and 3) according to optimal *k*-means clustering. Cluster analysis was performed with the Consensus Cluster Plus *R* package. The overall survival (OS) data of each cluster was calculated using the Kaplan–Meier method. The correlation between m6A-associated lncRNAs and clinical characteristics was analyzed according to TCGA database. The ESTIMATE algorithm was employed to estimate the tumor immune microenvironment.

### 5.4. m6A-Associated lncRNA Signature Construction

The prognostic m6A-associated lncRNAs were identified via univariate cox regression analysis. The prognostic signature was established via multivariate cox regression analysis. The risk scores of CRC patients were calculated by the following formula: Risk score = ∑Expi^*∗*^*β*i, where Expi represents the expression, and *β*i represents the coefficient of m6A-associated lncRNAs. The accuracy of the m6A-associated lncRNAs was assessed via the ROC curve analysis.

### 5.5. Statistical Analysis

All data were analyzed via by using *R* statistical software version 4.0.3. A p value less than 0.05 was statistically significant.

## Data Availability

The data that support our findings are openly available in TCGA (https://portal.gdc.cancer.gov/) repository.

## Abbreviations

lncRNAs: Long non-coding RNAs
CRC: Colorectal cancer
TCGA: The cancer genome atlas
GSEA: Gene set enrichment analysis
ROC: Receiver operating characteristic
M6A: N6-methyladenosine
FC: Fold change
CCP: Consensus cluster plus
AUC: Area under the curve
RNA seq: RNA sequencing.

## Supplementary Materials

Supplementary Materials Supplementary data_1: the information of the differentially expressed m6A-associated lncRNAs. Supplementary data_2: 14 m6A-associated lncRNA signatures were identified as independent prognostic factors after using multivariate Cox regression analysis.
